# Immunoadsorption Therapy for Patients with Dilated Cardiomyopathy and Heart Failure

**DOI:** 10.2174/157340308785160534

**Published:** 2008-08

**Authors:** Uichi Ikeda, Hiroki Kasai, Atsushi Izawa, Jun Koyama, Yoshikazu Yazaki, Masafumi Takahashi, Makoto Higuchi, Chang-Sung Koh, Keiji Yamamoto

**Affiliations:** 1 Department of Cardiovascular Medicine, Shinshu University Graduate School of Medicine, Nagano, Japan; 2 Department of Nephrology, Shinshu University Graduate School of Medicine, Nagano, Japan; 3 Biomedical Laboratory Sciences, Shinshu University Graduate School of Medicine, Nagano, Japan; 4 Division of Cardiovascular Medicine, Jichi Medical University, Tochigi, Japan

**Keywords:** Cardiomyopathy, adrenoreceptor, autoantibody, immunoadsorption, heart failure.

## Abstract

Several autoantibodies directed against cardiac cellular proteins including G-protein-linked receptors, contractile proteins and mitochondrial proteins, have been identified in patients with dilated cardiomyopathy (DCM). Among these autoantibodies, anti-β1-adrenoreceptor (AR) antibodies have long been discussed in terms of their pathogenetic role in DCM. Anti-β1-AR antibody-positive patients with DCM showed significant deterioration of NYHA functional class as well as reduced cardiac function compared to those in autoantibody-negative patients. Various studies with a limited number of patients indicate that the use of immunoadsorption to eliminate immunoglobulin G (IgG) significantly improves cardiac performance and clinical status in heart failure patients. Since removal of autoantibodies of the IgG3 subclass induces hemodynamic improvement and an increase in the left ventricular ejection fraction, antibodies belonging to IgG3 such as anti-β1-AR antibodies might play an important role in reducing cardiac function in patients with DCM. According to a recent report, however, the effect of hemodynamic improvement by immunoadsorption threapy was similar among patients who were positive and negative for anti-β1-AR antibodies, indicating that the beneficial effects of immunoadsorption might be not directly associated with the selective elimination of the β1-AR autoantibodies. Immunoadsorption therapy is a new therapeutic option for patients with DCM and heart failure, but further investigations are required to elucidate the specific antigens of cardiac autoantibodies responsible for the hemodynamic effects.

## INTRODUCTION

Dilated cardiomyopathy (DCM) is a progressive myocardial disease characterized by contractile dysfunction and ventricular dilatation. DCM is not a rare cause of congestive heart failure and the leading reason for heart transplantation world wide [1]. Although many different pathogenetic mechanisms and therapeutic treatments have been discussed, the ultimate answers to these questions are still lacking.

## AUTOANTIBODIES IN DCM PATIENTS

A variety of experimental studies suggest that alterations of the immune system might be involved in the pathogenesis of DCM [2]. A number of antibodies against various cardiac proteins have been identified in DCM, which can be divided into sarcolemmal proteins (e.g. myosin, actin, troponin and tropomyosin), mitochondrial enzymes (e.g. the ADP-ATP carrier, nicotinamide adenine dinucleotide dehydrogenase, ubiquinol-cytochrome-c reductase, lipoamide dehydrogenase and pyruvate dehydrogenase), heat-shock proteins (e.g. hsp70, hsp60 and hsc70) and surface receptors (e.g. β1-adrenoreceptors (AR) and muscarinic receptors [3-8]. Among these, the pathogenetic role of autoantibodies against β1-AR has been well investigated in experimental models [9-11] and human DCM [12-14]. The β1-AR is a 7-transmembarane G-protein-coupled receptor abundantly expressed on cardiomyocytes. Catecholamine binding to the β1-AR transmits an intracellular signal through a cAMP-dependent protein kinase A pathway that drives functional alterations in cardiomyocyte contractility.

Previously, Wallukat and his colleagues observed the immunoglobulin G (IgG) fraction in sera from DCM patients could induce a positive chronotropic effect on neonatal rat cardiac myocytes [15]. That effect was inhibited by the β1-blocking agent bisoprolol. It has also been reported that up to 33% of patients with DCM produce detectable circulating autoantibodies directed against epitope regions of the β1-AR [16], which bind to the second extracellular loop of β1-AR and cause a sustained stimulation of the cAMP-dependent protein kinase A pathway, and are finally associated with reduced cardiac function in those patients [13]. The pathogenic potential of β1-AR-specific autoantibodies was affirmed by recent studies in which recipient rodents developed DCM after passive transfer of β1-AR-specific antisera [17]. Jane-wit *et al*. [18] also reported that sustained agonism by β1-AR autoantibodies elicited caspase-3 activation, cardiomyocyte apoptosis, and DCM **in vivo**.

**Fig. (1):**
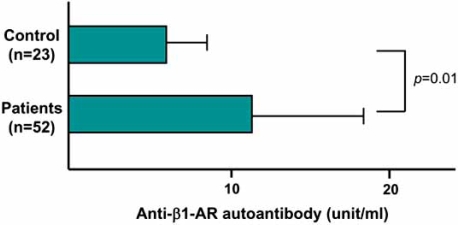
Comparison of plasma anti-β1-AR autoantibody levels in patients with chronic heart failure and control subjects.

An extremely high incidence of anti-β1-AR autoantibodies is also reported in end-stage DCM patients who require mechanical cardiac support [12]. In selective patients in whom cardiac function can be normalized by mechanical cardiac support, a gradual disappearance of autoantibodies accompanies the recovery. Other clinical evidence have documented that the presence of these autoantibodies is closely related to serious ventricular arrhythmias [19,20] and predicts increased cardiovascular mortality risk in DCM [21]. We screened for anti-β1-AR autoantibodies against the second extracellular loop of human β1-AR in 52 patients with chronic heart failure, and found that the mean values of autoantibodies in those patients were significantly higher than those in normal control subjects (Fig. **1**) [22]. Furthermore, during a follow-up of 3 years, patients with cardiac events had high anti-β1-AR autoantibody titers compared with patients without cardiac events. Thus, measurements of the β1-AR autoantibodies are important and useful for the management of chronic heart failure patients.

## IMMUNOADSORPTION THERAPY

**Fig. (2):**
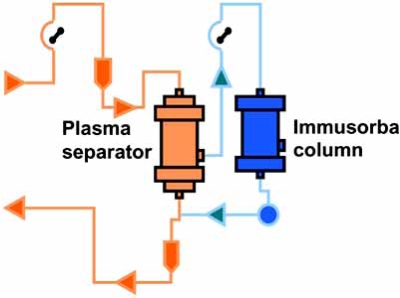
Immunoadsorption using the Immusorba column.

Removal of β1-AR autoantibodies with immunoadsorption (IA) is achieved by passing a patient’s plasma over columns that remove immunoglobulins (Fig. **2**). This IA for patients with DCM was first reported in an uncontrolled pilot study by Wallukat *et al*. [23], who showed that this technique efficiently removed circulating antibodies directed against the β1-AR. They also observed an improvement in NYHA functional class in those patients. That study was followed by other pilot studies that reported an improvement in short- and long-term hemodynamic effects in patients with heart failure, who were refractory to conventional medical therapy [24-26]. Dorffel *et al*. [24] performed IA on nine patients with DCM, left ventricular ejection fraction (LVEF) <25% on 5 consecutive days. During therapy, hemodynamic parameters were monitored with a Swan-Ganz thermodilution catheter. In those patients, a significant increase in cardiac output (from 3.7±0.8 to 5.5±1.8 L/min; p<0.01) and a significant decrease in mean arterial pressure and mean pulmonary arterial pressure was noted. Felix *et al*. [25] randomized 25 patients with DCM, LVEF<30% with evidence of β1-AR autoantibody to IA therapy *vs*. conventional therapy. The treatment group underwent monthly IA followed by immunoglobulin substitution for 3 months. IA therapy led to a significant decrease in β1-AR autoantibody levels. The increase in LVEF and improvement of NYHA class were significantly greater in the treatment group compared with those in the control group. Muller *et al*. [26] evaluated 34 patients with DCM with NYHA class II-IV, LVEF<29%, and evidence of elevated levels of β1-AR autoantibodies. The active treatment group of 17 patients underwent IA on 5 consecutive days. At 1 year, the treatment group experienced a significant increase in LVEF (0.22 to 0.38, p=0.0001) and improvement in NYHA class compared with no significant changes in LVEF in the control group. Staudt *et al*. [27] studied the effect of IA on plasma nt-BNP and nt-ANP levels in 15 patients demonstrating severe heart failure (LVEF<35%) due to DCM. Four courses of IA therapy were performed at monthly intervals until month 3. Three months after IA, patients demonstrated significant improvement in LVEF, reduction in left ventricular dimension and plasma nt-BNP levels. Those single-center, case-control studies suggested that IA therapy could improve NYHA class and LVEF in subjects with chronic DCM and heart failure. 

Previous studies used a variety of IA methods including specific anti-β1-AR antibody binding peptide columns (Coraffin®, Affina Immuntechnik) [28], nonspecific sheep anti-human IgG columns (Ig-Therasorb®, Plasmaselect) [29], or staphylococcal protein A-agarose columns (Immunosorba®, Fresenius HemoCare). The anti-β1-AR autoantibodies are included in IgG3, and Staudt *et al*. [30] reported a significantly improved cardiac index and LVEF for patients with DCM treated by IA with an anti-IgG column that removed significantly more IgG3 (89±3%) than in patients with DCM treated by IA with a protein A column that removed only 37±4% of IgG3. The protein A group did not achieve a significant increase in cardiac index or LVEF. Follow-up study of that series showed that IA with protein A columns with the addition of an improved treatment regime for IgG3 elimination could induce hemodynamic improvement in DCM patients [31]. Those studies indicated that the removal of IgG3 is essential to achieve therapeutic effects of IA to DCM. We have used tryptophan columns (ImmusorbaTR®, Asahi Kase Kuraray Medical) that contain cross-linked polyvinyl alcohol gel beads as the matrix to which the hydrophobic amino acid tryptophan is immobilized (Fig. **2**). It possesses nonselective physical features, but causes marked reduction of plasma levels of IgG3. In our protocol, plasma IgG and IgG3 levels dropped an average of 37% and 58% per single IA procedure, respectively.

Usually IA was followed by intravenous immunoglobulin (IVIG) to prevent infectious complications that might arise from inappropriate lowering circulating IgG levels [25]. Unlike most previous studies, however, Cooper *et al*. [32] did not substitute IVIG following IA for DCM patients to confirm the effect of IA. This was because IVIG at high doses can affect left ventricular function in chronic DCM [33], and has been associated with a significant rate of adverse events in subjects with autoimmune diseases [34]. They found that, even without IVIG substitution, IA for the treatment of DCM was associated with a significant improvement in the quality of life for up to 6 months after treatment. Global wall motion, as assessed by two-dimensional strain echocardiography, also showed a tendency towards improvement at 6 months. 

Although IA is a new therapeutic option for patients with DCM, the mechanism of left ventricular functional benefit from IA is not known. IgG adsorption removes not only anti-β1-AR-autoantibodies but also all other potentially pathogenic autoantibodies affecting the heart in this class of immunoglobulins. Mobini *et al*. [35] has reported that the effect of hemodynamic improvement during IA was similar among patients positive and negative for β1-AR autoantibodies. Their results suggest that the beneficial effects of IA are not directly associated with the selective elimination of β1-AR autoantibodies [36]. Schimke *et al*. [37] reported that a decrease in oxidative stress may be functionally important. In their study, three measures of oxidative stress, thiobarbituric acid-reactive substances, lipid peroxides, and antioxidized low density lipoprotein antibodies decreased significantly one year after selective IA of anti-β1-AR-autoantibodies with improvement of cardiac performance. 

## FUTURE DIRECTIONS

According to previous reports, the following questions remain to be resolved [38]: First, it will be important to identify the subsets of patients with DCM that will benefit the most from IA therapy, or determine whether patients with elevated levels of circulating autoantibodies (e.g., anti-β1-AR autoantibodies) should be studied. Second, the mechanism(s) underlying the action of IA have not yet been identified. Although studies have demonstrated decreases in circulating autoantibodies, it is not at all clear that a cause-and-effect relationship has been established. Third, the optimal strategy for IA has yet to be determined. Different investigators use different protocols and different immunoadsorbent devices. Moreover, the use of IVIG replacement in some of the IA protocols may improve the clinical status of patients with DCM [39], rather than neutralization of β1-AR autoantibodies. Finally, it is not clear from existing studies whether IA alone, which would be expected to modulate humoral immunity, will be sufficient over the long term, or whether it may be necessary to incorporate strategies that lead to suppression of cellular-mediated immunity as well. It is time to consider performing randomized clinical trials with IA in order to answer these questions.

## CONCLUSION

Measurements of anti-β1-AR autoantibodies may be helpful for the monitoring of clinical status in patients with DCM. IA therapy to eliminate autoantibodies is a new and promising therapeutic option for those patients. However, further studies are necessary to elucidate the specific antigens of cardiac autoantibodies as well as cellular mechanisms responsible for the observed functional effects.
